# Supplementary material for: Structure-based identification of bioactive compounds as trace amine-associated receptor 1 agonists for the therapeutic management of major depressive disorder
Source: PLoS One. 2025 Aug 5;20(8):e0327890. doi: 10.1371/journal.pone.0327890 (PMC12324101; doi:10.1371/journal.pone.0327890)
Supplement: S1 Fig — The figure was generated in PyMOL using the PDB structure with ID: 8JLO. The co-crystallized and docked Ulotaront molecules are superimposed, showing a root-mean-square deviation (RMSD) of 0.198 Å. (DOCX) [file pone.0327890.s001.docx]

*Supplementary Information*

**Structure-Based Identification of Bioactive Compounds as Trace Amine-Associated Receptor 1 Agonists for the Therapeutic Management of Major Depressive Disorder**

Abdelbaset Mohamed Elasbali^1,2^, Ahmed S. Ali^2,3,4^, Mohd Adnan^5^, Taj Mohammad^6^, Anas Shamsi^7^, and Md. Imtaiyaz Hassan^6,*^

^1^Department of Clinical Laboratory Science, College of Applied Medical Sciences-Qurayyat, Jouf University, Sakakah, Saudi Arabia.

^2^King Salman Center for Disability Research, Riyadh 11614, Saudi Arabia.

^3^Department of Physical Therapy and Health Rehabilitation, College of Applied Medical Sciences, Jouf University, Al-Qurayyat, Saudi Arabia.

^4^Department of Physical Therapy for Neurology and Neurosurgery, Faculty of Physical Therapy, Cairo University, Giza, Egypt.

^5^Department of Biology, College of Science, University of Ha'il, Ha'il, Saudi Arabia.

^6^Centre for Interdisciplinary Research in Basic Sciences, Jamia Millia Islamia, Jamia Nagar, New Delhi 110025, India.

^7^Centre of Medical and Bio-Allied Health Sciences Research, Ajman University, Ajman, United Arab Emirates.

**Dr. Md. Imtaiyaz Hassan, Ph.D., FRSB, FRSC.**

Professor

Centre for Interdisciplinary Research in Basic Sciences

Jamia Millia Islamia, New Delhi-110025, India

E-mail: [mihassan@jmi.ac.in](mailto:mihassan@jmi.ac.in)


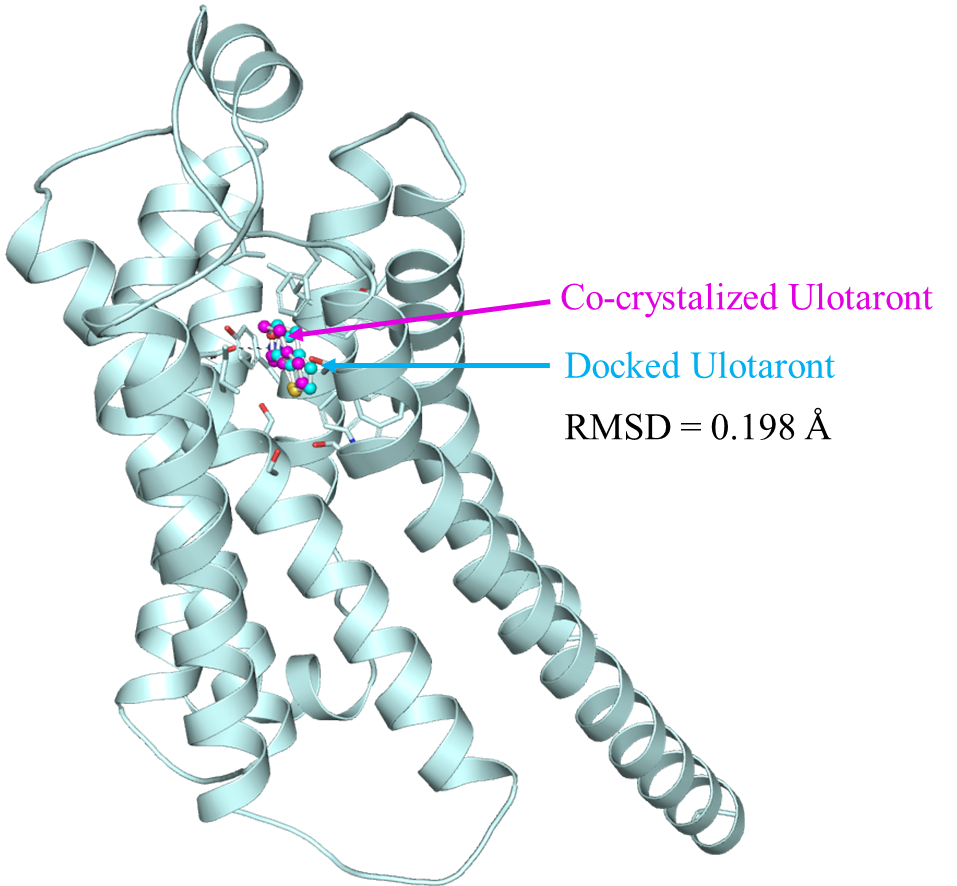


**Supplementary Fig. S1:** TAAR1 in complex with co-crystallized (magenta) and docked (cyan) Ulotaront. The figure was generated in PyMOL using the PDB structure with ID: 8JLO. The co-crystallized and docked Ulotaront molecules are superimposed, showing a root-mean-square deviation (RMSD) of 0.198 Å.
