# Supplementary material for: Structure-based identification of bioactive compounds as trace amine-associated receptor 1 agonists for the therapeutic management of major depressive disorder
Source: PLoS One. 2025 Aug 5;20(8):e0327890. doi: 10.1371/journal.pone.0327890 (PMC12324101; doi:10.1371/journal.pone.0327890)
Supplement: Attachment — Submitted filename: Response to Reviewers.docx [file pone.0327890.s003.docx]

**Reviewer #1:**

The manuscript presents a robust computational pipeline for identifying TAAR1 agonists, but addressing the above points will strengthen its rigor and translational potential. Emphasizing the need for experimental follow-up and discussing limitations will enhance the study’s credibility:

1. Mention the method that used to evaluate compounds through Lipinski's rule (please mention it in methodology)

**Response**: Thank you for your valuable suggestion. As advised, we have mentioned the use of Lipinski’s rule of five for the initial filtering of phytochemicals in the methodology section of the revised manuscript.

2. How to predict the binding site of TAAR1 (please, mention crucial amino acid residues that responsible for binding)

**Response**: We appreciate this insightful comment. The binding of TAAR1 is known as annotated at UniProt (https://www.uniprot.org/uniprotkb/Q96RJ0) based on multiple sources (PMID: 37935376, PMID: 37963465, PMID: 37935377). The manuscript has now been updated to clearly describe the key binding site residues of TAAR1, especially focusing on Asp103, Ser107, Tyr294, Trp264, Phe268, and others involved in hydrogen bonding and hydrophobic contacts. This information has been incorporated in the revised manuscript.

3. Why was InstaDock chosen over established tools like AutoDock or Glide for molecular docking? How was its reliability validated (e.g., through redocking of Ulotaront)

**Response**: Thank you for pointing this out. We have updated the Molecular Docking section (2.2) to justify our use of InstaDock, which offers an efficient and user-friendly graphical interface suitable for high-throughput screening. Its core algorithm is built on AutoDock Vina, ensuring comparable reliability. We have also performed the redocking analysis to validate the docking parameters and binding site selection, as recommended. The predicted binding poses are closely aligned with the experimentally determined crystallographic conformation of Ulotaront. The results have been mentioned in section 2.2 and Supplementary Fig. S1.

4. Could you elaborate on the structural features critical for TAAR1 binding? Are there opportunities for optimizing these scaffolds? (write discussion important SAR for TAAR1 binding)

**Response**: We thank the reviewer for highlighting this important point. Now, we have included a dedicated discussion within Section 3.4 on the SAR relevant to TAAR1 binding. We have emphasized the recurring presence of hydrophobic aromatic rings, H-bond donors near Asp103, and pi-pi stacking motifs, which appear critical for receptor activation. We have also discussed potential optimization strategies by modifying functional groups to enhance affinity and selectivity. Thanks!

**Reviewer #2:**

Having critically assessed your manuscript entitled “Structure-Based Identification of Bioactive Compounds as Trace Amine-Associated Receptor 1 Agonists for the Therapeutic Management of Major Depressive Disorder” (Manuscript ID: PONE-D-25-21674), and evaluated the reviewers’ comments, I would like to summarize the major points to be revised, including some suggestions to be considered for improvement. These edits serve to increase the clarity, scientific accuracy, and the overall quality of the manuscript prior to submission.

General Observations

The manuscript presents promising findings on phytochemical agonists targeting TAAR1 and their relevance to MDD.

The overall methodology is sound and comprehensive.

Improvements are needed in grammar, clarity, redundancy elimination, and data presentation for better scientific communication.

**Response**: We sincerely thank the reviewer for their overall positive assessment and valuable feedback. The manuscript has undergone a thorough grammatical revision, removal of redundancies, and clarification of ambiguous statements across all sections. Additionally, we have improved data presentation by breaking down complex descriptions into well-organized tables and figures during this revision.

Summary of Required Revisions by Section

1. Abstract

Correct grammatical issues and ensure complete sentence structure (e.g., Line 34–35).

Refine conclusion sentence for impact.

**Response**: We appreciate this suggestion. The Abstract has been revised for improved grammar, sentence clarity, and overall flow. The concluding sentence now emphasizes the translational significance and therapeutic promise of the identified phytochemicals as next-generation TAAR1 agonists.

2. Introduction

Remove unnecessary sentence structure and over-long sentences (e.g., Line 66, 89).

Clarify the role of TAAR1 in neuropsychiatry and why natural products are promising.

**Response**: We have carefully revised the Introduction to simplify and shorten complex sentences. The role of TAAR1 in neuropsychiatry has been articulated more clearly, emphasizing its advantages over monoaminergic targets. Furthermore, we highlighted the rationale for focusing on natural products, citing their historical success and pharmacological richness.

3. Materials and Methods

Define resolution and TAAR1 structure details (Line 117).

State docking box size and list all used software with URLs.

Mentioning known PRODRG limitations and PCA/FEL assumptions might be useful.

**Response**: Thank you for this comprehensive comment. We have made the following additions:

- Resolution and details of TAAR1 (PDB: 9JKQ) are now mentioned in Section 2.1.
- Docking box size and coordinates are described in Section 2.2.
- All tools and software with URLs have been listed where they are first mentioned.
- The known limitations of PRODRG and the assumptions made in PCA/FEL have been briefly discussed.

4. Results and Discussion

Quantitatively report data for RMSD, H-bonding, Rg, and SASA with SD as appropriate.

Breaking up complex interaction data into tables rather than wordy paragraphs may be a good idea.

**Response**: We are grateful for this practical suggestion. Accordingly, we have added quantitative results for RMSD, RMSF, Rg, SASA, and hydrogen bonding in Table 5 of the revised manuscript. Also, the protein-ligand interaction data have been summarized in Table 4 of the revised manuscript.

5. Conclusions

Correct typing errors and redundancy (e.g., "Peimisine and Peimisine").

Close with greater emphasis on translational potential and future work.

**Response**: Thank you for catching this typo. We have corrected the repetition and revised the Conclusion section to better highlight the translational potential of Bianthraquinone and Peimisine as TAAR1 agonists. We also added a note on future experimental validation and optimization of these scaffolds as part of a pipeline for antidepressant development.

6. Declarations

Declare consistent with "Funding: NONE" and the Acknowledgment thanking the King Salman Center.

I recommend that your manuscript be accepted

best regards

**Response**: We appreciate this observation. The funding statement has been updated to reflect accurate details. This is now consistently declared in both the Funding and Acknowledgments sections. Thanks!
